# Microfluidics as a Platform for the Analysis of 3D Printing Problems

**DOI:** 10.3390/ma12172839

**Published:** 2019-09-03

**Authors:** Rui Mendes, Paola Fanzio, Laura Campo-Deaño, Francisco J. Galindo-Rosales

**Affiliations:** 1 CEFT, Departamento de Engenharia Mecânica, Faculdade de Engenharia da Universidade do Porto, Rua Dr. Roberto Frias, 4200-465 Porto, Portugal; 2 Ultimaker B.V. Watermolenweg 2, 4191 PN Geldermalsen, The Netherlands; 3 CEFT, Departamento de Engenharia Química, Faculdade de Engenharia da Universidade do Porto, Rua Dr. Roberto Frias, 4200-465 Porto, Portugal; 4 Instituto de Ciência e Inovação em Engenharia Mecânica e Engenharia Industrial, Rua Dr. Roberto Frias, 400, 4200-465 Porto, Portugal

**Keywords:** microfluidics, 3D printing, back-flow, upstream vortices

## Abstract

Fused Filament Fabrication is an extrusion deposition technique in which a thermoplastic filament is melted, pushed through a nozzle and deposited to build, layer-by-layer, custom 3D geometries. Despite being one of the most widely used techniques in 3D printing, there are still some challenges to be addressed. One of them is the accurate control of the extrusion flow. It has been shown that this is affected by a reflux upstream the nozzle. Numerical models have been proposed for the explanation of this *back-flow*. However, it is not possible to have optical access to the melting chamber in order to confirm the actual behavior of this annular meniscus. Thus, microfluidics seems to be an excellent platform to tackle this fluid flow problem. In this work, a microfluidic device mimicking the 3D printing nozzle was developed, to study the complex fluid-flow behavior inside it. The principal aim was to investigate the presence of the mentioned back-flow upstream the nozzle contraction. As the microfluidic chip was fabricated by means of soft-lithography, the use of polymer melts was restricted due to technical issues. Thus, the working fluids consisted of two aqueous polymer solutions that allowed replicating the printing flow conditions in terms of Elasticity number and to develop a De–Re flow map. The results demonstrate that the presence of upstream vortices, due to the elasticity of the fluid, is responsible for the back-flow problem.

## 1. Introduction

Fused Filament Fabrication (FFF) or extrusion-based additive manufacturing, also known as *Fused deposition modeling (FDM)*, is one of the most widely used processes for rapid prototyping with common engineering plastics [1,2]. It is an extrusion deposition process, where a molten thermoplastic filament is heated in a liquefier and extruded through a nozzle depositing the material into layers forming a 3D part [3]. The most common thermoplastic materials used in this type of process are those having a low melting temperature and allowing a high productive rate, such as ABS (Acrylonitrile butadiene styrene), PLA (polylactic acid) and PC (Polycarbonate) [4]. Independently of the material used, real time monitoring has become one of the major challenges in the field of additive manufacturing [5], in order to improve the printing quality and to avoid the two main problems of FFF 3D printing: (1) nozzle clogging, which affects the quality of the printed parts since clogging conditions may vary during time [6] causing geometrical misalignments or a catastrophic failure [7]; and (2) the so-called *back-flow*, where the polymer melt can flow back up the annular region between the filament and the liquefier walls and cool outside of it, which could cause a major blocking problem. Gilmer et al. [8] developed a mathematical model aiming at predicting which material is prone to generate back-flow, based on several key properties, such as the filament diameter and the shear-thinning behavior of the material, although no experimental data backed-up this model.

It is well known from the late 1970s [9,10,11] that, when viscoelastic fluids flow through a pipe having a sudden axisymmetric contraction, there is a critical flow condition upon which sufficiently elastic fluids exhibit an instability in their flow that is not observed for purely viscous fluids. These pioneering results were experimentally confirmed by different authors for different viscoelastic fluids and contraction ratios [12,13,14,15]. Evans and Walters [16] also observed these upstream vortices when viscoelastic fluids flow through planar contraction and expansion geometries, with the contraction ratio and the elasticity of the fluids contributing for the enhancement of these vortices. even though this observation was performed at the macroscale, microfluidics provides a useful platform to achieve flow configurations where elastic forces become dominant and the inertial ones become residual [17,18,19,20,21,22,23]. This is possible because the characteristic length scale of the channels is smaller than 1 mm [24]. Additionally, assuming that the flow field in the 3D printing nozzle is axisymmetric and considering that the typical dimensions of the extrusion nozzle opening diameter in FFF lay in the order of hundreds of microns [25], it seems reasonable to use a microfluidic approach to analyze the back-flow problem.

In this study, we aimed at replicating several printing conditions in microfluidics. The printing conditions were defined in terms of the Deborah number (De), the Reynolds number (Re) and the Elasticity number (El). The cross section of the axisymmetric geometry of the printing nozzle was replicated in a planar microfluidic chip made of polydimethylsiloxane by using the soft lithography technique at different scales. The working fluids in the microfluidic experiments were selected so that the experiments provided the same Elasticity number as Polycarbonate in the printing conditions. Streakline photography allowed visualizing the flow upstream and downstream the contraction section for each flow configuration. Finally, a De–Re map was constructed. The FFF printing conditions are discussed in terms of the flow patterns observed in the microfluidic devices.

## 2. Materials and Methods

### 2.1. Real Printing Conditions (Prototype)

#### 2.1.1. Rheology Of Polycarbonate

The polycarbonate PC-MAX used in the 3D printing has the printing and mechanical characteristics presented in Table 1; however, the viscoelastic properties of the PC-MAX were not available. Considering that the Melt Flow Index (MFI) is defined as the weight of polymer extruded in ten minutes through a capillary of a specific diameter when applied a specific pressure and temperature, a polycarbonate with similar MFI value to the polycarbonate used in the 3D printing was considered as a reference to find a fluid analog for this study (Table 1). The rheological properties were extracted from the book “Handbook of Polycarbonate Science and Technology” [26]. Table 2 gives some information about the chosen polycarbonate here described as PC54.

**Figure 1:**
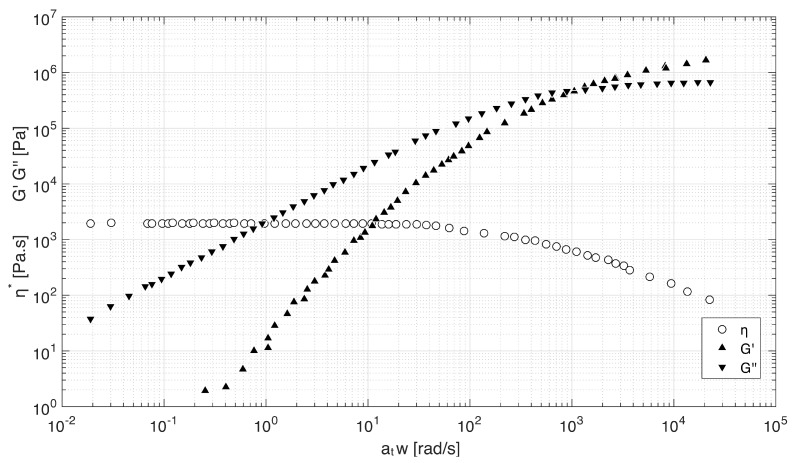
Master curve of dynamic properties of PC54: reference temperature of 275 ${}^{\circ }C$. Figure adapted from [26].

Figure 1 was also obtained from the book “Handbook of Polycarbonate Science and Technology” [26], which provides a master curve for $\eta^{*}$ and $G^{\prime }$ and $G^{\prime \prime }$. These master curves are obtained by applying the time-temperature superposition principle [28] to frequency sweep experiments within the linear viscoelastic regime at different temperatures. $a_{t}$ in Figure 1 is a function that accounts for the horizontal shift in ω. When materials smoothly shift, this indicates that the relaxation times associated with the material share the same temperature dependence. $\eta^{*}$ is calculated from $G^{\prime }$ and $G^{\prime \prime }$ as follows: [29].

(1) $$\eta^{*}=\frac{\sqrt{G^{\prime 2}+G^{\prime \prime 2}}}{\omega }$$

The Cox–Merz relationship [30] ($\eta \left(\dot{\gamma }\right)={\left|\eta^{*}\left(\omega \right)\right|}_{\dot{\gamma }}=\omega$), which holds for many polymeric systems including polycarbonate [26], allows substituting the measured linear viscoelastic $\eta^{*}\left(\omega \right)$ for the steady shear viscosity $\eta (\dot{\gamma })$. The marked shear thinning behavior can be observed, as the viscosity diminishes with an increasing shear rate. The shear rate at the nozzle can be defined by Equation (1):

(2) $$\dot{\gamma }=\frac{\bar{V_{c}}}{D_{c}/2}=\frac{8Q}{\pi D_{c}^{3}}$$

where *Q* is the flow rate and $D_{c}$ is the nozzle diameter. The flow rate is a printing parameter and the range between 10 and 50 mm${}^{3}$/s was considered in this study. Each flow rate produces a different shear rate at the contraction which results in different values of viscosity and ultimately different values of Re. The shear rate can be directly read in the graph of Figure 1. It was possible to perform a straight read from the figure since a printing temperature of 275 ${}^{\circ }C$ was considered. If any other temperature were to be considered, it would be necessary to introduce a shift scale $a_{t}$ in order to perform a correct read of the graph.

The frequency sweep test allows observing the time-dependent response of a sample within the linear viscoelastic range. Short-term behavior is provided by the response at high frequencies, while low frequency gives information about the long-term behavior. The relaxation time can be obtained from the crossover of $G^{\prime }$ with $G^{\prime \prime }$ in Figure 1 [31] and shown in Table 3:

(3) $$\lambda =\frac{1}{\omega }$$

where λ is the relaxation time and ω is the frequency in Hz.

The last parameter necessary to calculate the dimensionless numbers is the density of the molten polymer at the considered temperature which can be expressed as shown in Equation (4) [32] (Table 3):

(4) $$\rho =\frac{10^{3}}{\exp(-0.307+1.86\times 10^{-5}T^{3/2})}$$

#### 2.1.2. Dimensions of the 3D Printing Nozzle

**Figure 2:**
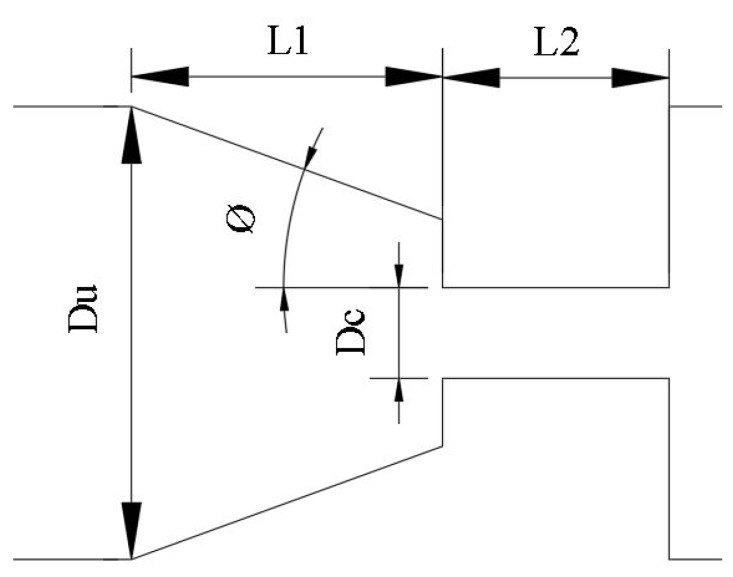
Main dimensions of the 3D printer nozzle.

Figure 2 shows the design of the 3D printing nozzle, where all key dimensions are indicated in Table 4.

### 2.2. Planar Microfluidic Nozzle (Model)

#### 2.2.1. Microchips Design and Fabrication

**Figure 3:**
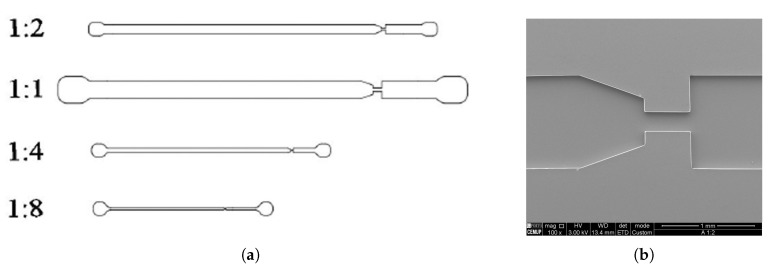
(**a**) Top-view of the design of the 2D microfluidic nozzles with different scale ratios; and (**b**) top-view of a PDMS microchannel (1:2) taken by means of Scanning Electron Microscopy.

The design of the microfluidic nozzle was done in 2D by using a Computer-Aided-Design software (AutoCAD, Autodesk^®^, California, US). Up to four different scale ratios were considered, namely 1:1, 1:2, 1:4 and 1:8, as can be observed in Figure 3. Each microchannel possesses its own inlet and outlet ports. Between the inlet and the outlet, there is a contraction that replicates the cross section of the 3D printing nozzle and a sudden expansion to simulate the expansion of the polymeric material when leaves the nozzle and interacts with the atmosphere.

Polydimethylsiloxane (PDMS) is probably the most commonly used elastomer in microfluidic devices for many reasons, such as simple fabrication procedure, strong sealing to a wide variety of materials, low cost, biocompatibility, chemical inertness, low toxicity, ease of manipulation, durability, etc. [33]. The quality of the microfluidic channels is directly related to the master. In this study, the master used to cast molding the PDMS was fabricated using photolithography. Ultraviolet light was used to transfer the geometry of the channels from a photomask to a photosensitive substrate (SU-8 wafer) [34,35].

The PDMS channels were fabricated using a two-part Sylgard ^®^ 184 PDMS polymer kit (Dow Corning, Midland, MI, USA), mixed to weight ratio of 10:1 (pre-polymer:curing agent) and degassed in a vacuum chamber until all the air bubbles were removed. Before pouring the mixture of PDMS and curing agent, the SU-8 molds were exposed to a gas surface treatment with TMCS (Trimethylchlorosilane) for about 1 h in order to facilitate the demolding process. After pouring the PDMS mixture onto the SU-8 mold, air bubbles were removed under vacuum and then cured in an 80 ${}^{\circ }C$ pre-heated oven for 30 min. The crosslinked PDMS was gently cut with a scalpel and carefully peeled off their molds. Next, the microchannels’ inlet and outlet ports were made with a stainless steel tip mounted in a plastic syringe. A Nordson precision tip with an inner diameter of 0.51 mm and an outer diameter of 0.82 mm was used. Finally, the PDMS were bonded onto a 50 mm × 75 mm microscope glass by using a plasma cleaner [36]. The real dimensions for the PDMS channels in each mold were determined from SEM images; the average values and standard deviations are provided in Table 5.

#### 2.2.2. Streak Line Photography

The fluid-flow was characterized by streak line photography, which consists of recording particles displacements over a period of time, allowing a qualitative analysis of the flow pattern [37]. The optical setup consisted of an inverted epi-fluorescence microscope (DMI 5000 M, Leica Microsystems GmbH) equipped with a sensitive monochromatic CCD camera (DFC350 FX, Leica Microsystems GmbH); a light source (100 W mercury lamp); 5×, 10×, 20× or 40× magnification objective lens (Leica HCX PL Fluotar L CORR, numerical aperture NA = 0.40); and a filter cube (Leica Microsystems GmbH, excitation filter BP 530,545 nm, dichroic 565 nm and barrier filter 610–675 nm). The microscopy images were acquired and analyzed afterwards using the Leica Application Suite Software, or LAS (v3.5.0, Leica Microsystems). The flow rate at the inlet was controlled by means of a neMESYS low pressure syringe pump (Cetoni GmbH 14:1), which operates on a motor that pushes the syringe at a constant speed; in order to ensure pulsation-free dosing, a set of Hamilton syringes (10 μL up to 2.5 mL) was used depending on the required flow rate. The syringes were connected to the PDMS channels by means of Tygon tubing with a 0.44 mm inner diameter and Nordson precision tips with an inner diameter of 0.33 mm. The outlet was left open to the atmosphere. The working fluids were seeded with 1 μm fluorescent tracer particles (Nile Red, Molecular Probes, Invitrogen, Ex/Em: 520/580 nm). To guarantee a good visualization, small concentrations of particles (40 and 90 ppm) were added to the analog fluid. Instead of using Sodium Dodecyl Sulfate (SDS) to minimize adhesion of fluorescent tracer particles (hydrophobic particles) to the channel walls, a surface treatment to the PDMS with oxygen plasma was applied to each microchannel in order to turn them hydrophilic [38].

#### 2.2.3. Working Fluids

Polymer solutions were used as working fluids, due to the practical impossibility of using polymer melts in microfluidic channels made out of PDMS. Aqueous solutions of Polyacrylamide (PAA) with a molecular weight Mw = 18$\times 10^{6}$ g·mol${}^{-1}$ were used instead of Polycarbonate at different weight concentrations (1000 and 10,000 ppm) in de-ionized water. The mixing process was developed with magnetic stirrers at low speeds for three days in order to ensure the homogeneity of the sample while preventing any mechanical degradation of the polymer molecules [39]. The samples were sealed with a parafilm tape to prevent partial evaporation of the solvent during the mixing process.

#### 2.2.4. Rheological Characterization

##### Shear Rheometry

A steady shear rheological characterization of the PAA solutions was performed by means of a control stress rotational rheometer (Anton Paar, model Physica MCR301). A 50 mm diameter plate/plate (PP) geometry with a gap of 0.1 mm was used in order to obtain reliable results at higher shear rates [40]. The steady-state viscosity curves were performed for a shear rate sweep from 0.1 to 100,000 1/s. All experiments were performed at 20 ${}^{\circ }C$ and at least three times to ensure the reproducibility. The window of reliable data was established between the two limiting viscosity lines: (1) the minimum torque line, which provides the minimum value of viscosity from which the results are not affected by the resolution of the rheometer (Equation (5) [40])

(5) $$\eta_{min}=\frac{2M_{0}}{\pi R^{3}\dot{\gamma }}$$

where $M_{0}$ is the torque resolution of the rheometer ($10^{-7}$ Nm), *R* the geometry radius and $\dot{\gamma }$ the shear rate; and (2) the onset of secondary flows at high shear rates, because even without a turbulent flow the primary shear flow can be overlaid by a secondary flow, which creates an extra dissipation leading to an increase in torque (Equation (6))

(6) $$\eta_{sec}>\frac{H^{3}\rho \dot{\gamma }}{12\hat{R}R}$$

where *H* is the gap, ρ is the density of the fluid and $\hat{R}$ is a parameter ($\hat{R}=0.5$).

##### Extensional Rheometry

The characterization of the PAA solutions under uniaxial extensional flow was performed in the Capillary Breakup Extensional Rheometer (Haake CaBER 1, Thermo Scientific). The fluid sample was set between two parallel plates separated by a gap $h_{0}$, being the bottom plate stationary and the top one imposing a step strain deformation, leading to a non-equilibrium situation resulting in a filament thinning process. The filament thinning process was recorded using a high speed video camera (Photron FASTCAM Mini UX100), at 1000 fps. Each set of images was analyzed by means of the Matlab Image Processing Toolbox, which allowed determining the time evolution of the minimum diameter $D_{mid}\left(t\right)$. The characteristic shapes of $D_{mid}\left(t\right)$ for each fluid represent different material properties and the relaxation time of the liquids can be determined from the exponential decay of the filament diameter [19]. The evolution in the midpoint profile is governed by a force balance, which can be described by:

(7) $$D_{mid}\left(t\right)=D_{0}{\left(\frac{GD_{0}}{4\sigma }\right)}^{1/3}exp\left(-\frac{t}{3\lambda }\right)$$

where λ is the characteristic relaxation time of the polymeric liquid.

All experiments were performed at 20 ${}^{\circ }C$ and at least five times to ensure the reproducibility.

### 2.3. Dimensionless Numbers

To ensure a proper replication of the real flow conditions in the microfluidic channel (model), it was necessary to guarantee that Re and De were as similar as possible to the prototype. To that end, the dimensionless numbers for the real case (prototype) were analyzed. The contraction of the nozzle can be seen as a cylindrical pipe, thus $Re_{prototype}=\frac{\rho \bar{V}D_{c}}{\eta }$, where ρ is the polymeric fluid density, $\bar{V}$ is the mean velocity, $D_{c}$ the contraction diameter and η is the viscosity of the polymer melt (evaluated at each corresponding shear rate ($\dot{\gamma }$). Considering that the mean velocity is related to the cross section area ($A=\frac{\pi D_{c}^{2}}{4}$) and the flow rate (*Q*) by $Q=\bar{V}A$, then

(8) $$Re_{prototype}=\frac{4\rho Q}{\pi D_{c}\eta }$$

It is also necessary to take into account the elastic effects, which are represented by $De_{prototype_{max}}=\lambda \dot{\epsilon }$, where:

(9) $$\dot{\epsilon }\approx \frac{\delta V_{x}}{\delta x}\approx \frac{V_{c}-V_{u}}{L}$$

being $\dot{\epsilon }$ the extension rate, $V_{c}$ the maximum velocity at the contraction, $V_{u}$ the maximum velocity upstream of the contraction and *L*=$L_{1}$+$L_{2}$ (Figure 2). The maximum velocity can be related to the mean velocity with a coefficient *k* as:

(10) $$V_{max}=k\bar{V}$$

Then, by combining Equations (9) and (10), the extension rate at the centerline can be calculated as [41]:

(11) $$\dot{\epsilon }=\frac{4Q}{\pi L}\left(\frac{k_{c}}{D_{c}^{2}}-\frac{k_{u}}{D_{u}^{2}}\right)$$

where $D_{u}$ is the diameter upstream, $D_{c}$ is the diameter of the contraction and $k_{u}$ and $k_{c}$ are the coefficients relating the maximum velocity and the mean velocity upstream the contraction and at the contraction, respectively, which can be calculated as shown by Deplace et.al [42] as:

(12) $$k=\frac{v_{max}}{\bar{v}}=\frac{\pi^{4}}{64\sum_{n=1,3,5,...}^{\infty }\frac{1}{n^{4}}\left(1-\frac{2a}{n\pi b}\tanh\left(\frac{n\pi b}{2a}\right)\right)}-\frac{\pi \sum_{n=1,3,5,...}^{\infty }\frac{1}{n^{3}}{\left(-1\right)}^{\frac{n-1}{2}}\frac{1}{\cosh(\frac{n\pi b}{2a})}}{2\sum_{n=1,3,5,...}^{\infty }\frac{1}{n^{4}}\left(1-\frac{2a}{n\pi b}\tanh\left(\frac{n\pi b}{2a}\right)\right)}$$

where *b* is the width and *a* the depth of the channel. For pipes of circular cross-section, which is the case of the 3D printing nozzle, k=2. Thus, using the maximum velocity methodology, $De_{prototype}$ can be written as:

(13) $$De_{prototype}=\frac{8\lambda Q}{\pi L}\left(\frac{1}{D_{c}^{2}}-\frac{1}{D_{u}^{2}}\right)$$

In the case of the microchannels, the cross section is a rectangular one, thus Reynolds number is defined as $Re_{model}=\frac{\rho \bar{V}D_{h}}{\eta }$, and $D_{h}=\frac{4A}{P}=\frac{2w_{c}h}{w_{c}+h}$ is the hydraulic diameter of the microchannel, where $A=w_{c}h$ is the cross-section area and $P=2(w_{c}+h)$ is the wetted perimeter of the microchannel, being $w_{c}$ the contraction width and *h* the depth of the microchannel. Then, Reynolds number inside the microchannel takes the form:

(14) $$Re_{model}=\frac{2\rho Q}{(w_{c}+h)\eta }$$

In addition, De changes due to the rectangular shape of the channels compared to the case of the prototype:

(15) $$De_{model}=\frac{\lambda Q}{hL}\left(\frac{k_{c}}{w_{c}}-\frac{k_{u}}{w_{u}}\right)$$

where $w_{u}$ is the width of the channel upstream the contraction and $k_{u}$ and $k_{c}$ are the coefficients relating the maximum velocity and the mean velocity upstream the contraction calculated by Equation (12) [42]. Table 6 shows the values of the k’s coefficients for each model at microscale.

Table 7 shows the values of Re and De for the case of the prototype calculated from Equations (8) and (13), respectively, for each given flow rate value.

The dynamic similarity must be satisfied to ensure that the ratios of forces acting at the corresponding points in the model and prototype are the same in magnitude. However, it is a common experience in fluid mechanics problems to face difficulties in satisfying this condition for all dimensionless numbers, as in aerodynamics where the perfect matching experimentally both Reynolds and Mach numbers is impossible to be achieved. Such is the case here for Re and De and the model is designed on the basis of equating the dimensionless number dominating the problem, i.e., El. It is interesting to note that although El does not depend directly on the flow rate, its value can almost double or cut in half depending on each flow condition. This can be explained by the variation of viscosity due to the shear-thinning behavior of the polymer melt. As the velocity of printing decreases, the viscosity increases substantially, lowering Re to the point that it is a larger decrease than De, thus promoting a higher El; in other words, it enhances the elastic effects and lowers the inertial ones. Based on this, it was decided to analyze the flow pattern inside the nozzle at different El values since it seems to correspond to different printing speeds.

## 3. Results and Discussion

### 3.1. Rheological Behavior of the Polymer Solutions

**Figure 4:**
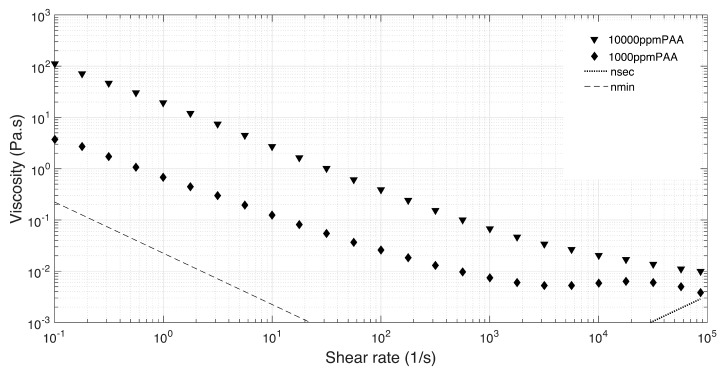
Viscosity curves for PAA solutions.

Figure 4 shows the steady shear viscosity curves for the aqueous solutions of PAA at different concentrations. As expected, the shear thinning behavior becomes more pronounced as the polymer concentration is larger.

These samples were also characterized under extensional flow by using the CaBER, in order to obtain the relaxation time of the fluids (Table 8).

The other parameter necessary is the density of the PAA solutions, which was calculated by means of the equation provided by Saravanan et al. [43], where the density can be expressed by the equation:

(16) $$\rho =A+B_{1}w+B_{2}w^{2}+B_{3}w^{3}$$

where ρ is density of the solution at the desired temperature; A, $B_{1}$, $B_{2}$, and $B_{3}$ are coefficients of the polynomial that varies with temperature; and *w* is mass fraction of PAA in the solution. The density was calculated for a temperature of 20 ${}^{\circ }C$, as presented in Table 9.

Finally, to calculate the dimensionless numbers, it is necessary to determine the shear at the microfluidic contraction by adapting Equation (1) with $A=hw_{c}$:

(17) $$\dot{\gamma }=\frac{2Q}{w_{c}^{{}^{2}}h}$$

The dimensionless numbers for 1000 and 10,000 ppm of PAA water solutions to be used for the fluid-flow analysis of the printing conditions in the microfluidic model of the nozzle are presented in Table 10 and Table 11, respectively.

**Figure 5:**
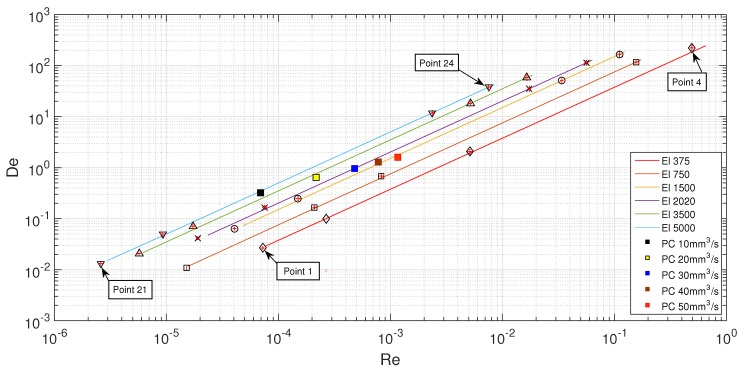
De–Re flow map.

As can be seen in Table 10 and Table 11, it is possible to achieve the same El in any of the microchannels; however, it is not possible to exactly replicate both Re and De of the polycarbonate at the same time, which makes it impossible to fully replicate the printing conditions. However, if we analyze more carefully both tables, it is possible to see that we have a list of points around the same printing condition, which potentiates the creation of a De–Re flow pattern map to collect a full picture of the possible flow conditions, as shown in Figure 5. Thus, six. different values of El were selected, in order to mimic a wide printing conditions beyond the limits of the real printing speeds shown in Table 7. Inside each El, four points were chosen, with two of them at lower Re and De and two at higher Re and De, in an attempt to represent all the different flow conditions possible. With this configuration, it would be possible to understand the source of the back-flow inside the FFF printer based on the flow pattern observed in the planar microfluidic nozzle.

The selected points were then represented as dots, as shown in Figure 5, with Re, De and El. The square points represent different real printing conditions with PC (Table 12), while the remaining points correspond to the flowing conditions in the microfluidic channels with the PAA solutions (Table 13). It is possible to achieve all of working points by combining different scale geometries, different fluids and different flow rates.

### 3.2. Fluid-Flow Characterization

**Figure 6:**
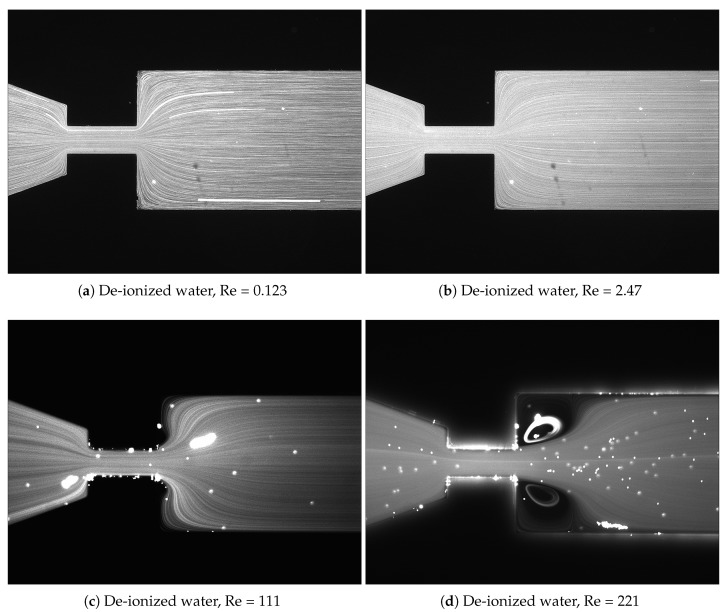
Flow pattern for a Newtonian fluid at different values of Re. The flow direction is from left to right.

To understand the influence of the elasticity of the fluid on the fluid-flow pattern when flowing through the planar microfluidic nozzle, experiments with de-ionized water at different Re values were performed. As expected (Figure 6), the Newtonian fluid flow exhibits a laminar profile at low Re. Above a critical Re, the flow transforms into an asymmetric flow pattern with generation of vortices downstream of the contraction, as shown in the work of Campo-Deano et al. [19]. This phenomenon occurs due to an inefficient dissipation of kinetic energy as the fluid decelerates as it passes to the expansion area, creating a recirculation zone due to a pressure loss [41].

**Figure 7:**
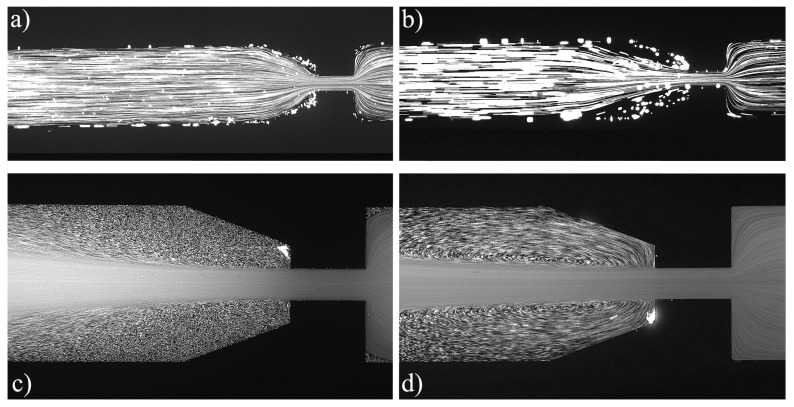
Flow pattern for PAA solutions at El∼375 and different flow conditions: (**a**) Point 1; (**b**) Point 2; (**c**) Point 3; and (**d**) Point 4 (Table 13). The flow direction is from left to right.

**Figure 8:**
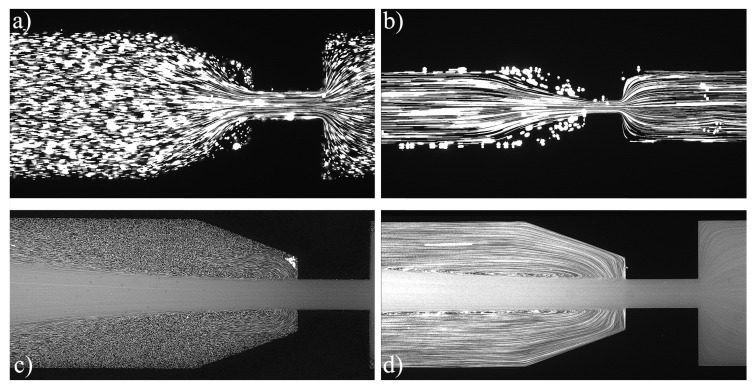
Flow pattern for PAA solutions at El∼750 at different flow conditions: (**a**) Point 5; (**b**) Point 6; (**c**) Point 7; and (**d**) Point 8 (Table 13). The flow direction is from left to right

**Figure 9:**
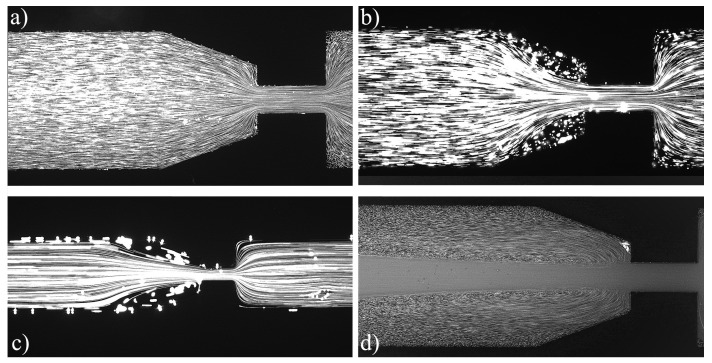
Flow pattern for PAA solutions at El∼1500 and different flow conditions: (**a**) Point 9; (**b**) Point 10; (**c**) Point 11; and (**d**) Point 12 (Table 13). The flow direction is from left to right.

**Figure 10:**
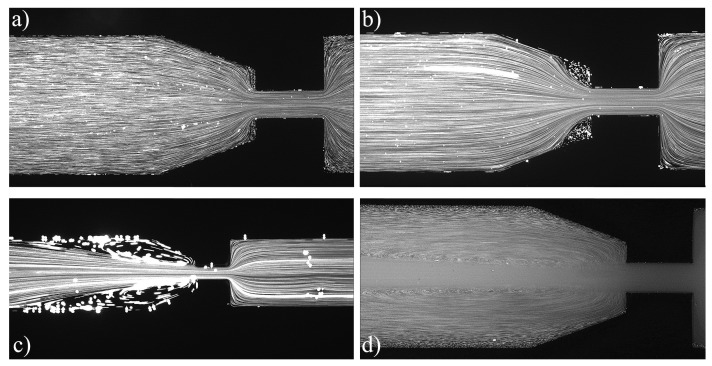
Flow pattern for PAA solutions at El∼2020 and different flow conditions: (**a**) Point 13; (**b**) Point 14; (**c**) Point 15; and (**d**) Point 16 (Table 13). The flow direction is from left to right.

**Figure 11:**
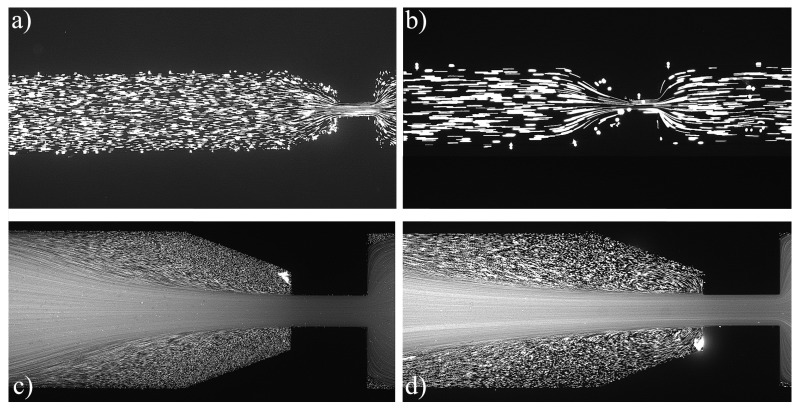
Flow pattern for PAA solutions El∼3500 and different flow conditions: (**a**) Point 17; (**b**) Point 18; (**c**) Point 19; and (**d**) Point 20 (Table 13). The flow direction is from left to right.

**Figure 12:**
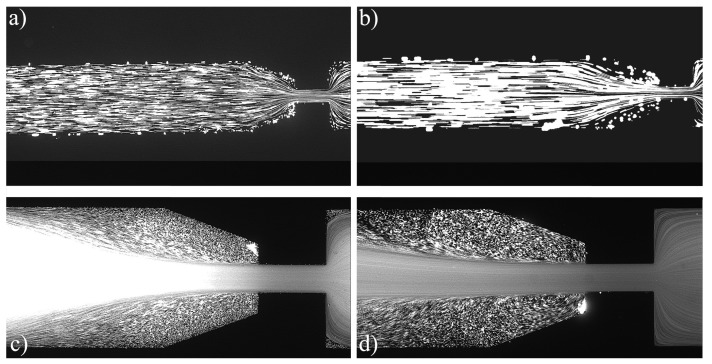
Flow pattern for PAA solutions El∼5000 and different flow conditions: (**a**) Point 21; (**b**) Point 22; (**c**) Point 23; and (**d**) Point 24 (Table 13). The flow direction is from left to right.

For the PAA solutions, since they are viscoelastic fluids, symmetric vortices develop upstream of the contraction due to elastic effects, which is an absolute opposite to the behavior of Newtonian fluids [19]. Figure 7, Figure 8, Figure 9, Figure 10, Figure 11 and Figure 12 exhibit the flow pattern of the PAA samples through the microfluidic device with the flow conditions defined in Table 13.

**Figure 13:**
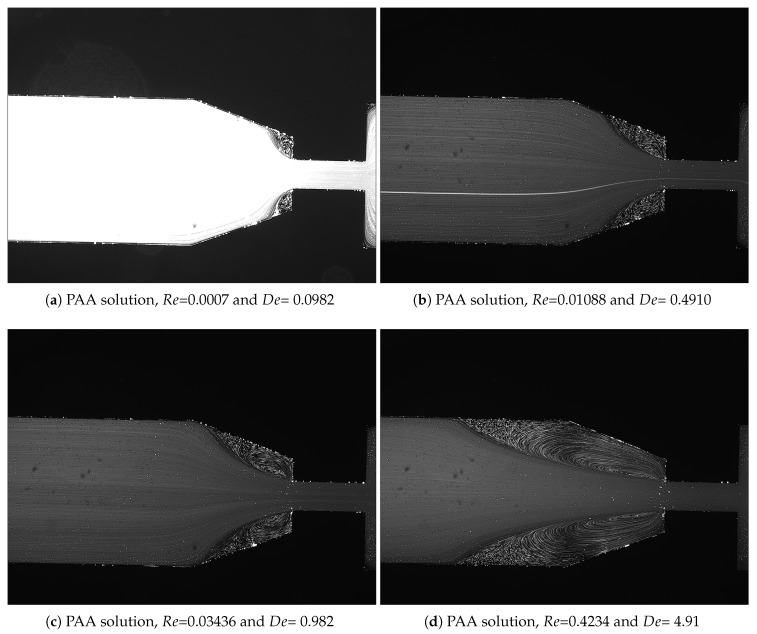
Vortex generation and growing inside a microchannel. The flow direction is from left to right.

According to Figure 7, Figure 8, Figure 9, Figure 10, Figure 11 and Figure 12, it was possible to identify three different flow patterns: At lower Re and De, the fluid maintains attached to the walls without a visible instability of vortex formation, revealing a Newtonian-like behavior for small Re. When Re and De were increased, a second regime could be identified, where the fluid detached from the walls of the microchannel and an incipient vortex formation with the fluid rotating very slowly could be distinguished (e.g., Figure 8c). When additional experiments were performed at higher Re and De values, the formation of upstream vortices were more evident and a strong vortex enhancement was observed by further increasing the Re and De (Figure 13), which is consistent with the results obtained by Rothstein and McKinley [14], Galindo-Rosales et al. [22] and Sousa et al. [44]. Moreover, the flow within the vortex is quite slow when compared with the flow at the centerline of the microchannel, which supports the idea of the incipient vortex formation due to the elasticity of the fluid.

For higher Re and De, we can see preferential central path and larger vortices due to the progressive enhancement of elastic effects, not only showing a bigger length but also its center dislocating further left, away from the contraction as shown in Figure 9d.

In terms of actual printing conditions, we can analyze Points 14 and 22 (Figure 10b and Figure 11b), since these points are the ones closer to the printing conditions, namely polycarbonate with a flow rate of 30 mm${}^{3}$/s and 10 mm${}^{3}$/s, respectively. It is possible to observe a funnelled flow path, but, as shown by Figure 13, this is caused by upstream vortices. However, the printing conditions have a slightly higher De and Re, which would further potentiate the elastic effects resulting in even larger vortices compared to the ones observed in those figures. These vortices are responsible for two main effects: The first one is the formation of a funnelled path, as discussed above, which leads to an increased velocity at the centerline, resulting in a greater flow rate to the one programmed. The second one is the back-flow effect, resulting in a reflux upstream the nozzle between the piston and the liquefier walls. Larger vortices lead to more material located near the walls that is not extruded, creating an even more significant effect.

To get the working points of Table 14 during the printing conditions without changing the design of the nozzle, it is necessary to change the working polymer. Using Equations (8) and (13), it is possible to determine the viscosity, relaxation time of a polycarbonate and flow rate necessary to replicate these values in the real geometry. Table 15 presents three possible polymers in a conceptual way for each point presented in Table 14, by varying the viscosity, relaxation time and flow rate needed to achieve the desired Re and De.

By using the book “Handbook of Polycarbonate of Science and Technology” [26] as a source of available polycarbonate materials, not all of them can be used in practice. For example, Polymers 1, 2, 4, 7 and 10 would exhibit a relatively slow flow rate, which translates into a low printing speed, resulting in longer waiting time to achieve a final piece. Thus, the more plausible solution would be to re-design numerically the geometrical configuration of the nozzle in order to minimize the size of the vortex upstream the contraction even for relatively high flow rates.

## 4. Conclusions

**Figure 14:**
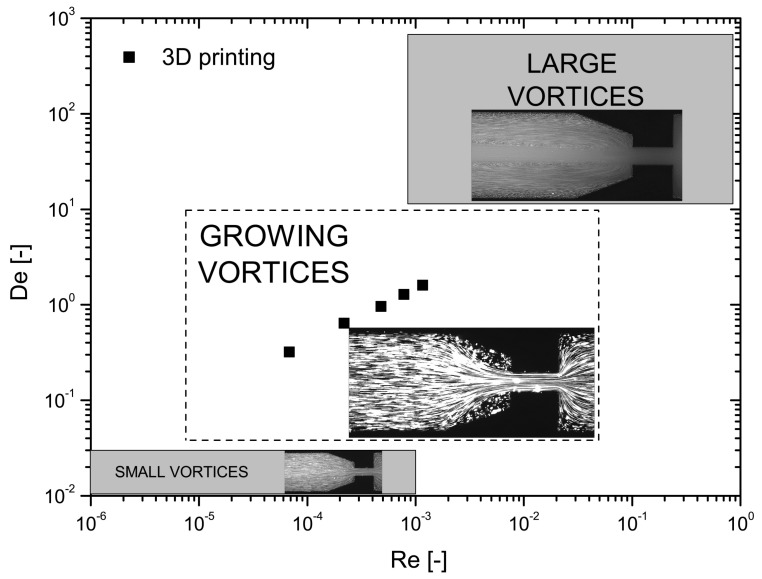
De–Re map indicating the different flow patterns.

The results of the flow visualizations inside four planar microfluidic nozzles with different scales were analyzed. The fabrication of these microchannels is a complex process and required a considerable amount of time to produce an error free mask. The different scales gave an important flexibility in terms of replicating the printing conditions for different El values, being possible to create a De–Re map (Figure 14) of flow patterns for this geometrical configuration. Three different flow pattern zones were distinguished: at lower Re and De, it was possible to observe a laminar profile, very similar to a Newtonian fluid-flow at low Re, where the fluid stayed attached to the walls of the microchannel without any disturbance; as the elastic effects increased, a second zone was visible, where the flow detached from the walls upstream of the contraction and the typical printing conditions were located; and, finally, if the Re and De were further increased, large vortices promoted a preferential central flow path. Considering the dimensions of the vortices upstream the contraction, it is possible to conclude that they may be responsible for the back-flow problem in the 3D printer.

As expected, it was possible to observe the formation of vortices upstream of the contraction, due to the elastic effects of the analog fluids, leading to a volume of fluid rotating at lower velocity than the extruded one. This enhances heat transfer through the nozzle walls and near the contraction, which may alter the *in-line* rheological measurements provided by Coogan and Kazmer [45] if the vortices reach the pressure port and polymer solidifies there blocking the measurement. Eventually, they could even lead to a formation of solid pieces, promoting clogging. Furthermore, the vortex creates a zone of under-extruded material, which can accumulate near the walls up to the point it escapes between the filament and liquefier, resulting in a back-flow effect that leads to a catastrophic failure in a 3D printing process.

This work constitutes a first microfluidics approach to elucidate the physics behind the main problems in a printing process when using viscoelastic polymers. In future works, a wider range of analog fluids should be studied, not only to further complete the flow pattern map, but also to replicate the exact conditions of the printer and to discover the critical Re and De values that potentiate a laminar flow. Another approach could be creating different geometry configurations and analyze if any one of them retards or even prevents the vortex generation. Finally, using all of this information, it would be possible to try to replicate numerically the extruding process inside the 3D printing in terms of flow pattern and heat transfer.

## Figures and Tables

**Table 1:** Technical information of the polycarbonate used in the 3D printing [27].

Density	1.18–1.20 (g/cm${}^{3}$ at 21.5 ${}^{\circ }C$)
Glass Transition Temperature	113 (${}^{\circ }C$)
Melt Index	6–8 (g/10 min) at 260 ${}^{\circ }C$
Nozzle temperature (printing)	
Printing Speed	30–50 (mm/s)
Young Modulus	2048 ± 66 (MPa)
Tensile strength	59.7 ± 1.8 (MPa)
Elongation at break	12.2 ± 1.4 (%)

**Table 2:** PC 54 properties [26].

PC 54	
Inherent Viscosity (dl/g)	0.54
MFI (g/10 min)	7.4
Mw (g/mol)	60,600
Mn (g/mol)	24,400
Mw/Mn	2.5

**Table 3:** Crossing frequency and relaxation time of PC54.

$\omega_{\mathrm{crossing}}$ [Hz]	182.48
Relaxation time (λ) [s]	5.48 × $10^{-3}$
Density (ρ) [kg/m${}^{3}$]	1070.7

**Table 4:** Main dimensions of the 3D printing nozzle.

$D_{u}$	2000 μm
$D_{c}$	400 μm
$L=L_{1}+L_{2}$	2373 μm
ϕ	20 ${}^{o}$

**Table 5:** Dimensions of the microchannels obtained from SEM analysis.

Scale Ratio	$W_{u}\pm \mathrm{SD}$ (μm)	$W_{c}\pm \mathrm{SD}$ (μm)	L±SD (μm)	$\theta \pm \mathrm{SD}{(}^{\circ })$
1:1	2030 ± 40	401 ± 6	2390 ± 40	20 ± 1
1:2	1000 ± 14	202 ± 6	1200 ± 13	20 ± 1
1:4	504 ± 2	106 ± 2	595 ± 7	20 ± 1
1:8	255 ± 2	57 ± 3	301 ± 9	20 ± 1

SD. Standard Deviation.

**Table 6:** *k* coefficients for each microfluidic channel.

Ratio	$K_{u}$	$K_{c}$
1:1	1.524	1.628
1:2	1.549	1.773
1:4	1.601	1.992
1:8	1.715	2.096

**Table 7:** Dimensionless numbers for the 3D printing nozzle printing a PC54 polymer.

Q [mm ${}^{3}$ /s]	Re	De	El=De/Re
10	6.79 × $10^{-5}$	0.322	4740
20	2.18 × $10^{-4}$	0.644	2890
30	4.78 × $10^{-4}$	0.966	2020
40	7.79 × $10^{-4}$	1.29	1620
50	1.16 × $10^{-3}$	1.61	1390

**Table 8:** Relaxation time for 1000 and 10,000 ppm PAA samples.

PAA Sample	Relaxation Time (λ±SD) [s]
1000 ppm	0.139 ± 0.003
10,000 ppm	1.57 ± 0.16

SD = Standard Deviation.

**Table 9:** Density for the PAA solutions at 20 ${}^{\circ }C$.

	*w*	*A*	$B_{1}$	$B_{2}$	$B_{3}$	ρ [g/cm${}^{3}$]
1000 ppm	0.001	0.9982	0.3196	0.1435	−0.1493	998.5
100,00 ppm	0.01					1001

**Table 10:** Dimensionless numbers for 1000 ppm PAA water solutions.

Scale	$\dot{\gamma }$ [1/s]	Re	De	El
1:1	0.3474	2.858 × $10^{-6}$	0.005350	1873
1:2	0.5806	4.905 × $10^{-6}$	0.009875	2013
1:4	2.253	1.903 × $10^{-5}$	0.04141	2176
1:8	8.475	7.538 × $10^{-5}$	0.1672	2218

**Table 11:** Dimensionless numbers for 10,000 ppm PAA water solutions.

Scale	$\dot{\gamma }$ [1/s]	Re	De	El
1:1	218.1	0.01824	35.01	1920
1:2	603.3	0.05720	116.5	2021
1:4	2158	0.3209	692.4	2158
1:8	1.107 $\times \;10^{4}$	1.280	2828	2209

**Table 12:** Dimensionless numbers of several real printing conditions with PC.

	Re×10−5	De	El
PC 10 mm${}^{3}$/s	6.789	0.322	4643
PC 20 mm${}^{3}$/s	21.85	0.644	2955
PC 30 mm${}^{3}$/s	47.78	0.966	2021
PC 40 mm${}^{3}$/s	77.91	1.288	1653
PC 50 mm${}^{3}$/s	115.9	1.610	1389

**Table 13:** Dimensionless numbers of several flowing conditions of PAA solutions in the microfluidic channels.

	(Re±SD)·$10^{-3}$	De±SD	El±SD
Point 1	0.072 ± 0.018	0.027 ± 0.002	366.9 ± 22.7
Point 2	0.267 ± 0.009	0.098 ± 0.007	368.0 ± 21.1
Point 3	5.125 ± 0.229	2.063 ± 0.201	402.6 ± 26.5
Point 4	493.7 ± 12.20	223.4 ± 26.7	452.6 ± 53.0
Point 5	0.015 ± 0.004	0.011 ± 0.001	714.2 ± 44.1
Point 6	0.210 ± 0.006	0.166 ± 0.011	789.8 ± 40.4
Point 7	0.830 ± 0.037	0.683 ± 0.066	822.9 ± 54.2
Point 8	156.5 ± 3.9	117.3 ± 14.0	749.4 ± 87
Point 9	0.043 ± 0.001	0.066 ± 0.004	1545 ± 79
Point 10	0.150 ± 0.007	0.249 ± 0.024	1664 ± 110
Point 11	33.980 ± 0.840	50.50 ± 6.04	1486 ± 174
Point 12	111.2 ± 3.7	166.9 ± 20.5	1501 ± 172
Point 13	0.019 ± 0.001	0.041 ± 0.003	2176 ± 111
Point 14	0.075 ± 0.003	0.167 ± 0.016	2218 ± 146
Point 15	17.46 ± 0.43	35.1 ± 4.19	2008 ± 236
Point 16	56.56 ± 1.90	114.5 ± 14.1	2025 ± 232
Point 17	0.005 ± 0.001	0.020 ± 0.001	3900 ± 199
Point 18	0.017 ± 0.001	0.070 ± 0.001	4132 ± 272
Point 19	5.183 ± 0.129	18.10 ± 2.16	3491 ± 409
Point 20	16.58 ± 0.56	58.20 ± 7.17	3511 ± 403
Point 21	0.003 ± 0.001	0.015 ± 0.001	5084 ± 260
Point 22	0.009 ± 0.001	0.048 ± 0.001	5372 ± 353
Point 23	23.74 ± 0.58	118.4 ± 1.4	4987 ± 584
Point 24	7.549 ± 0.253	37.90 ± 4.66	5014 ± 575

SD = Standard Deviation.

**Table 14:** Experimental points and their corresponding dimensionless numbers exhibiting a laminar profile.

	Re	De	El
Point 1	7.240 $\times \;10^{-5}$	2.657 $\times \;10^{-2}$	366.9
Point 5	1.515 $\times \;10^{-5}$	1.082 $\times \;10^{-2}$	714.2
Point 17	4.826 $\times \;10^{-6}$	1.882 $\times \;10^{-2}$	3900
Point 21	2.592 $\times \;10^{-6}$	1.317 $\times \;10^{-2}$	5084

**Table 15:** Different polymer that can create a laminar profile inside a FFF 3D printing nozzle with the current geometrical configuration.

	$\eta \;\cdot \;10^{4}$ [Pa·s]	λ [ms]	*Q* [mm${}^{3}$/s]	Re·10−6	De	$\mathrm{El}\;\cdot \;10^{3}$
Polymer 1	0.006	3.300	1.39	77.6	0.0295	0.38
Polymer 2	0.049	0.410	11.1	77.7	0.0293	0.38
Polymer 3	0.131	0.152	30.0	77.8	0.0293	0.38
Polymer 4	0.030	1.320	1.39	15.8	0.0118	0.75
Polymer 5	0.240	0.165	11.1	15.8	0.0118	0.75
Polymer 6	0.648	0.061	30.0	15.8	0.0118	0.75
Polymer 7	0.076	2.430	1.39	6.20	0.0217	3.50
Polymer 8	0.610	0.304	11.1	6.21	0.0217	3.50
Polymer 9	1.650	0.112	30.0	6.20	0.0216	3.49
Polymer 10	0.155	1.710	1.39	3.05	0.0153	5.01
Polymer 11	0.124	0.214	11.1	3.05	0.0153	5.01
Polymer 12	3.350	0.079	30.0	3.05	0.0152	5.00
